# Identification and Analysis of Biomarkers Associated With Lipid Metabolism and Ferroptosis in Ulcerative Colitis

**DOI:** 10.1155/jimr/9283449

**Published:** 2026-05-13

**Authors:** Xinmei Zhang, Xiufang Cui

**Affiliations:** ^1^ Department of Gastroenterology, Nanjing First Hospital, Nanjing Medical University, Nanjing, 210006, China, njmu.edu.cn

**Keywords:** biomarkers, ferroptosis, lipid metabolism, ulcerative colitis

## Abstract

**Background:**

Mounting evidence shows that lipid metabolism and ferroptosis contribute to ulcerative colitis (UC), but the mechanism remains unclear. This study aimed to identify related biomarkers, clarify their roles in UC, and provide insights into optimized therapies.

**Methods:**

UC transcriptome, lipid metabolism, and ferroptosis‐related gene (FRG) data were analyzed. Biomarkers were screened via differential expression analysis, consensus clustering, Venn, and machine learning, with expression validation. Receiver operating curve (ROC) analysis assessed predictive efficacy; functional enrichment, molecular regulatory, and immune infiltration analyses were performed. Real‐time PCR verified candidate biomarkers in clinical samples.

**Results:**

Two biomarkers (acyl‐CoA synthetase ligases 4 [*ACSL4*] and prostaglandin‐endoperoxide synthase 2 [*PTGS2*]) were identified that distinguished UC from control samples. They may involve hematopoietic cell lines, cytokine–cytokine receptor interaction, and MALAT1 binding hsa‐miR‐576‐5p/hsa‐miR‐503‐5p. Notably, 27 differentially infiltrated immune cells were found (*p*  < 0.05), with CD56dim natural killer cells negatively correlating with *ACSL4*/*PTGS2*. Both genes were significantly upregulated in the UC clinical samples.

**Conclusion:**

*ACSL4* and *PTGS2* are lipid metabolism‐ and ferroptosis‐related biomarkers of UC, laying a foundation for clinical treatment.

## 1. Introduction

Ulcerative colitis (UC), one of the two main inflammatory bowel disease (IBD) types, has shown a marked upward trend in global incidence over recent decades [1]. Pathologically, UC is characterized by chronic inflammation of the rectal and colonic mucosa, resulting in symptoms such as abdominal pain, bloody stools, and urgent defecation. Repeated disease flare episodes and associated complications markedly impair patients’ daily functioning and quality of life [2, 3]. UC pathogenesis involves complex interactions among genetic susceptibility, environmental triggers (e.g., diet and microbiota), and dysregulated immune responses [4, 5], although precise mechanisms remain unclear. UC remains incurable to date. Pharmacotherapies for achieving disease remission include 5‐aminosalicylic acid agents, corticosteroids, immunosuppressive agents (e.g., thiopurines), biologics (e.g., anticytokine and anti‐integrin agents), and small‐molecule compounds such as Janus kinase inhibitors and sphingosine‐1‐phosphate receptor modulators [1, 2]. However, ~30% and 40% of patients are refractory to the therapeutic regimens currently in clinical use [6, 7] due to the complex UC pathology that involves diverse signaling molecules and that different cytokines predominate, depending on disease stage and patient characteristics. Therefore, a single drug often fails to comprehensively target all inflammatory signaling pathways, triggering a therapeutic ceiling [8]. This highlights the need for deeper mechanistic insights. Identifying novel biomarkers and therapeutic targets is crucial to improving disease prediction and enabling precision medicine approaches to overcome the UC therapeutic ceiling [3, 4].

As essential components of biological membranes, lipids play vital functions in diverse cellular processes such as signal transduction [9, 10], cell differentiation [11], and apoptosis [12]. Dysregulated lipid metabolism has been implicated in the pathogenesis of multiple inflammatory disorders, including UC, with altered lipid signaling likely underpinning some of these challenges. In UC, dysregulated lipid metabolism can lead to the aberrant production of signaling molecules, such as eicosanoids (e.g., prostaglandins and leukotrienes), which act as key inflammation mediators. These molecules modulate immune cell recruitment, epithelial barrier integrity, and cytokine responses, thereby exacerbating disease heterogeneity and treatment resistance [9, 13, 14]. Understanding lipid signaling pathways could thus provide insights into personalized therapeutic strategies. These multifaceted challenges highlight the urgent need for deeper mechanistic insights. Lipid metabolism dysregulation markedly contributes to UC development, as the high regeneration rate of mucosal epithelial cells is closely related to the high lipid synthesis rate, which ensures epithelial cell membrane integrity. Recent studies have explored the lipid metabolism–UC correlation. The discovery of abnormal lipid metabolism biomarkers and UC alleviation through lipid metabolism regulation has provided a new therapeutic perspective [9, 15–18].

Iron deficiency compromises mucosal barrier integrity, promotes dysbiosis, and influences local inflammation [19]. Defined as an iron‐dependent form of regulated cell death, ferroptosis is triggered by uncontrolled peroxidation of phospholipids, which is also a regulated necrosis process and a response to tumor suppression [20]. Xu et al. [21] reported that ferroptosis participates in intestinal epithelial cell demise in UC, with NF‐κB p65 markedly contributing to ferroptosis inhibition. These findings underscore ferroptosis as a potential therapeutic target for UC treatment [21]. Interestingly, defined as an iron‐dependent cell death modality driven by the overwhelming buildup of membrane lipid peroxides, ferroptosis presents a novel therapeutic avenue for disease intervention—one that can be harnessed by targeting lipid metabolism to either promote or inhibit this cell death process [22]. Against this backdrop, exploring how lipid metabolism governs ferroptosis in UC is essential to advancing mechanistic insights and treatment options.

Most previous studies have investigated acyl‐CoA synthetase ligases 4 (*ACSL4*) or prostaglandin‐endoperoxide synthase 2 (*PTGS2*) in UC in isolation [23–25], often focusing solely on their individual roles in either lipid metabolism or ferroptosis. The fundamental novelty of this study lies in its integrative computational approach that systematically identifies and validates the coregulatory signature of *ACSL4* and *PTGS2* at the intersection of lipid metabolism and ferroptosis in UC by employing a combination of consensus clustering, machine learning algorithms (least absolute shrinkage and selection operator [LASSO] and support vector machine‐recursive feature elimination [SVM‐RFE]), and comprehensive analyses of the immune microenvironment and regulatory networks. To gauge the screening capacity of the target biomarkers for the study samples, receiver operating curve (ROC) analysis was conducted. Following this, gene set enrichment analysis (GSEA), immune infiltration assessment, targeted drug prediction, and molecular regulatory network analysis were performed to explore the functional mechanisms through which the candidate biomarkers contribute to UC pathogenesis. The findings derived from these analyses establish a rigorous framework for enhancing our comprehension of UC’s molecular etiology, accelerating the development of novel treatment regimens, and unlocking new avenues for personalized UC therapy.

## 2. Materials and Methods

### 2.1. Data Acquisition

Two UC‐related gene expression datasets—GSE87466 and GSE38713—were downloaded from the public Gene Expression Omnibus (GEO) database (http://www.ncbi.nlm.nih.gov/geo/). Among these, 87 UC and 21 control mucosal tissue samples were retained in GSE87466 (platform: GPL13158). Notably, 30 UC and 13 control mucosal tissue samples were acquired from GSE38713 (platform: GPL570) [26, 27]. By applying the keyword lipid metabolism for database querying, we acquired 882 lipid metabolism–related genes (LMRGs) from the Metabolic Signature Database (https://www.gsea-msigdb.org/gsea/msigdb). Based on their involvement in key lipid metabolic processes and related signaling pathways, these genes were further categorized into several functional subgroups, including cholesterol metabolism, fatty acid metabolism, and sphingolipid/ceramide signaling (Table S1). In addition, 88 ferroptosis‐related genes (FRGs) were obtained from the literature [28] (Table S2); 12 genes mediating ferroptosis regulation through lipid metabolism (LM‐FRGs) were collated from the available scientific literature: *HRAS*, *NRAS*, *KRAS* [29], *GPX4* [30], *SLC7A11* [31], *FSP1* [32], *DHODH*, *CHAC1*, *PTGS2*, *ACSL4* [33], *ALOX12*, and *iPLA2*β [34].

### 2.2. Consensus Clustering Analysis

To identify disease subtypes associated with lipid metabolism–regulated ferroptosis, the ConsensusClusterPlus package (V 1.66.0) [35] was used. Utilizing the expression levels of the 12 identified LM‐FRGs, a consensus clustering analysis was performed on UC samples from the GSE87466 dataset. To perform t‐distributed stochastic neighbor embedding (t‐SNE) dimensionality reduction analysis on UC samples using the R package Rtsne, we aimed to visualize the distribution characteristics of distinct molecular subtypes (obtained via consensus clustering) in a low‐dimensional space and evaluate the separation degree between subtypes. The main parameters for the t‐SNE analysis were set as follows: 2‐dimensional embedding (dims = 2), perplexity = 10, and maximum number of iterations (max_iter) = 500.

This approach was adopted to ensure that the selected genes not only differentiated UC from controls but also reflected intrinsic molecular heterogeneity within UC subtypes.

### 2.3. Differential Expression Analysis

Differentially expressed genes (DEGs) between UC and control specimens in the GSE87466 dataset (designated as DEGs1) and those between distinct subtypes (designated as DEGs2) were identified using the limma package (v3.58.1) based on a linear model framework integrated with empirical Bayes methodology for variance stabilization and statistical inference [36], with the thresholds set at adjusted *p*‐value < 0.05 and |log_2_ fold change (FC)| > 1. For graphical visualization, volcano plots were generated via the ggplot2 package (V 3.5.1; http://webofscience-clarivate-cn-s.webvpn.zju.edu.cn:8001/wos/alldb/full-record/WOS:000285969600026). Heat maps were constructed using the ComplexHeatmap package (V 2.21.1) [37].

### 2.4. Identification and Functional Analysis of Candidate Genes

The overlapping genes among DEGs1, DEGs2, LMRGs, and FRGs were defined as candidate genes, which were identified using the VennDiagram package (V 1.7.3; https://CRAN.R-project.org/package-VennDiagram). To elucidate the biological functions of these candidate genes, we employed the ClusterProfiler package (V 4.10.1) [38] to perform Gene Ontology (GO) and Kyoto Encyclopedia of Genes and Genomes (KEGG) enrichment analyses, with a statistical significance set at an adjusted *p*‐value < 0.05. We performed GO enrichment analysis covering three fundamental categories—biological processes (BPs), cellular components (CCs), and molecular functions (MFs)—to functionally annotate the identified genes. Correspondingly, KEGG analysis was utilized to uncover the dominant metabolic and signaling pathways in which these genes are implicated.

### 2.5. Biomarker Selection and ROC Analysis

Biomarkers of lipid metabolism–regulated ferroptosis were identified using LASSO logistic regression implemented in the R package glmnet (version 4.1.1). A binomial family parameter was specified for binary outcome modeling, with *α* = 1 imposed to enforce the LASSO penalty for variable selection. Model stability was enhanced by setting a random seed (set.seed(2)) and employing 10‐fold cross‐validation (nfolds = 10) to determine the optimal regularization parameter λ. Cross‐validation selected both λ.min (minimum mean cross‐validated error) and λ.1se (largest λ within 1 standard error of λ.min); however, λ.min was chosen for the final model construction due to its superior predictive accuracy. The candidate genes in the GSE87466 dataset underwent SVM‐RFE via the caret package (V 6.0.93; https://CRAN.R-project.org/package=caret), with recursive elimination integrated as a core step. Candidate biomarkers were retrieved by taking the intersection of the distinct gene subsets extracted from the LASSO regression and SVM‐RFE, utilizing the VennDiagram package (V 1.7.3) for this overlap analysis.

To delineate candidate biomarker expression levels in the GSE87466 and GSE38713 datasets and screen valid biomarkers, the rstatix package (V 0.7.2; https://CRAN.R-project.org/package=rstatix) was designed to perform Wilcoxon tests, comparing expression disparities of candidate biomarkers between UC and control specimens in the two datasets (*p*  < 0.05). Final biomarkers were defined as genes that displayed consistent and significant upregulation in UC samples relative to controls across both datasets (*p*  < 0.05). ROC curve analysis validated the discriminative performance of the identified biomarkers in the GSE87466 dataset, with an area under the curve (AUC) > 0.7 indicating a cutoff for acceptable classification ability [39, 40].

### 2.6. GSEA of Biomarkers

We used GSEA to explore the potential biological functions of biomarkers associated with UC progression. Spearman’s correlation between individual biomarkers and other genes was evaluated using the psych package (V 2.4.3) [41], leveraging UC and control sample data from the GSE87466 dataset. Thereafter, the correlation coefficients were ordered in a descending manner, and GSEA was executed on the prioritized gene set via the ClusterProfiler package (V 4.10.1) [38] to identify enriched BPs. The c2.cp.kegg.v7.4.symbols.gmt gene set, sourced from the GSEA database (http://www.gsea-CCigdb.org/gsea/CCigdb), was harnessed as the KEGG pathway background set for performing GSEA (*p*.adjust < 0.05 and |normalized enrichment score (NES)| > 1).

### 2.7. Immune Microenvironment Analysis

Analyzing the extent of infiltration of immune cells can provide valuable insights into UC progression. The ssGSEA algorithm using the GSVA package (v 1.50.0) [41] was utilized to compute the enrichment levels of 28 immune cell populations with infiltrative properties in each sample of the GSE87466 dataset [42]. Notably, we used the Wilcoxon test to further compare immune cell enrichment score differences between the UC and control cohorts in GSE87466 (*p*  < 0.05). Moreover, Spearman’s correlation analysis was performed using the psych package (V 2.4.3) [41] to unravel the correlative links between differentially expressed immune cells and biomarkers, adhering to the thresholds of |*R*| > 0.3 and *p*  < 0.05.

### 2.8. Regulatory Mechanisms of Biomarkers

To further dissect noncoding RNAs and transcription factors (TFs) potentially governing the biomarkers, key miRNAs (i.e., biomarker‐associated candidates) were inferred using the multiMiR package (V 1.24.0) [43] with a confidence threshold > 0.5. This approach leverages multiMiR’s integration of multiple miRNA–mRNA prediction databases (e.g., DIANA‐microT, ElMMo, MicroCosm, miRanda, miRDB, PicTar, PITA, and TargetScan). After retrieving all predicted interactions and tallying counts per database, we selected a database with moderate prediction volume and broad coverage amid interdatabase variability as the candidate interaction set for downstream ceRNA network construction. LncRNAs that interact with these key miRNAs were subsequently identified from the starBase database (http://starbase.sysu.edu.cn/), and a Sankey diagram was created using the ggplot2 package (version 3.5.1) to delineate the regulatory interplay among biomarkers, key miRNAs, and lncRNAs. Conclusively, biomarker–related TFs were forecasted via the JASPAR database embedded in the NetworkAnalyst platform (https://www.networkanalyst.ca/).

### 2.9. Analysis of Drug Forecasts

To uncover potential therapeutic candidates for UC, the Comparative Toxicogenomics Database (CTD; https://ctdbase.org/) was leveraged to predict UC‐directed drugs, with a reference count cutoff set at ≥2. Additionally, the DGIdb (https://www.dgidb.org/)—a specialized drug–gene interaction repository—was used to anticipate drugs that interact with the biomarkers. The associative relationships of drugs with dual specificity for UC and *PTGS2*, as predicted by the CTD and DGIdb databases, were visualized using a network diagram constructed with Cytoscape (V 3.9.1) [44].

### 2.10. Verification of Potential Biomarkers in Clinical Samples

We compared target gene expression levels between the disease and control groups to evaluate their biomarker potential for UC. Tissue samples from the disease group were obtained from inflamed areas of the colon mucosa, whereas those from the control group were obtained from normal colon mucosa. Written informed consent was obtained from all patients before biopsy collection, and the local ethics committees approved the study protocol. Total RNA was extracted from all samples; cDNA synthesis was subsequently performed using the HiScript III 1st Strand cDNA Synthesis Kit (supplemented with a gDNA wiper reagent). Real‐time PCR assays were performed to amplify the target and reference genes for each sample. Triplicate reactions were conducted for each experimental condition, while quantitative data processing was accomplished using the 2^−ΔΔCt^ approach. When performing real‐time quantitative PCR, the volume of each sample added was 2 µL. However, RNA concentration quantification error and reverse transcription efficiency variation prevented identical cDNA content in the 2 µL aliquot from each sample. To correct this difference, we used an internal reference (with a basically constant expression level among the different samples) for correction. Based on the original detection results of real‐time PCR, the relative quantification calculation formula of 2^−ΔΔCt^ was used—that is, *F* = 2 ^− [(the average Ct value of the target gene in the test group − the average Ct value of the housekeeping gene in the test group) − (the average Ct value of the target gene in the control group − the average Ct value of the housekeeping gene in the control group)]^—to calculate the relative quantitative results of the target genes for each sample; that is, the differences in the mRNA transcription levels of the target genes among the various samples compared to the control group. An independent samples *t*‐test was executed to dissect discrepancies in gene expression across the two groups (*p*  < 0.05).

### 2.11. Statistical Analysis

Bioinformatic analyses were executed with the R programming language (V 4.2.2), wherein pairwise group comparisons of data were accomplished using the Wilcoxon test. A threshold of *p*  < 0.05 was applied to denote statistical discernibility, except where explicitly specified otherwise.

## 3. Results

### 3.1. Differential Expression Analysis Between Samples and Subtypes

In the GSE87466 dataset, 1085 DEGs1 were detected between UC and control samples, comprising 750 upregulated and 335 downregulated transcripts in UC specimens. The volcano plot annotated the top 10 DEGs1 with the highest log2FC (both upregulated and downregulated), and the heat map depicted the expression characteristics of these selected genes (Figure 1A,B).

Figure 1Differential expression analysis between samples and subtypes. (A) Volcano plot of DEGs1 between UC (*n* = 87) and control (*n* = 21) samples in the GSE87466 dataset. Differential expressions were determined using the limma package (adjusted *p*  < 0.05 and |log_2_FC| > 1). The top 10 upregulated and downregulated genes were labeled. (B) Heat map showing the expression profiles of the top 10 DEGs1 across all 108 samples. (C) Consensus clustering matrix for *k* = 2. (D) Consensus cumulative distribution function (CDF) plot. (E) Relative change in the area under the CDF curve. (F) t‐SNE plot validating the two subtypes. (G–H) Volcano plot and heat map of DEGs2 between the two identified UC subtypes.

**Figure:**
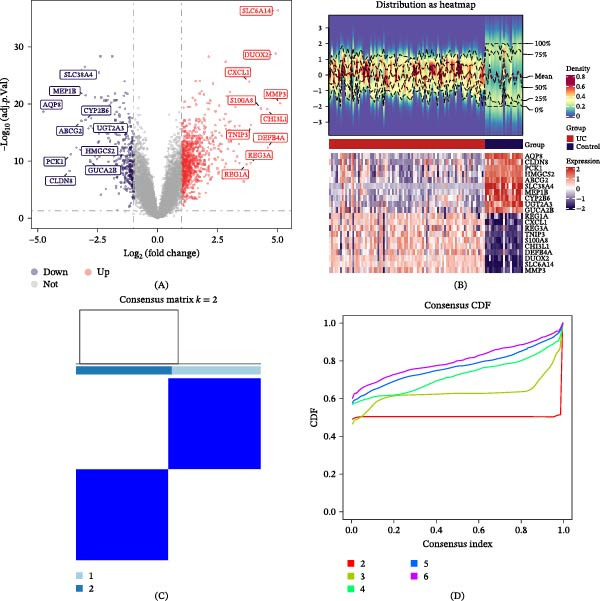

**Figure:**
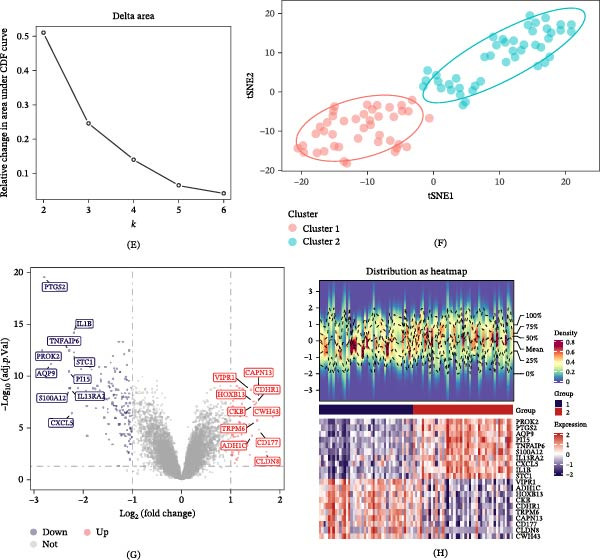

Consensus clustering analysis was performed on the GSE87466 UC samples using the expression patterns of 12 LMRGs. Stability in the subtype number was detected at *k* = 2 (Figure 1C,D), with the relative fluctuations in the area under the cumulative distribution function (CDF) curve maintaining consistency between *k* = 2 and *k* = 6 (Figure 1E). The optimal cluster number (*k* = 2) was determined by minimizing the CDF error, thus enhancing subtype classification robustness. On this basis, the UC samples were categorized into two subtypes (i.e., subtype 1 and subtype 2), with t‐SNE analysis confirming this classification validity (Figure 1F).

A total of 204 DEGs2 were identified between the two subtypes, with 53 upregulated DEGs2 and 151 downregulated DEGs2 in the subtype 1 samples. Sorted by log_2_FC, the top 10 upregulated and downregulated DEGs were marked on the volcano plot, and the heat map was employed to exhibit the expression dynamics of these genes (Figure 1G,H). This intersection strategy ensured that the candidate genes captured both general UC‐associated changes and subtype‐specific variations, thereby providing a more comprehensive pool for biomarker discovery.

### 3.2. Identification Analysis and Functional Enrichment of Three Candidate Genes

**Figure 2:**
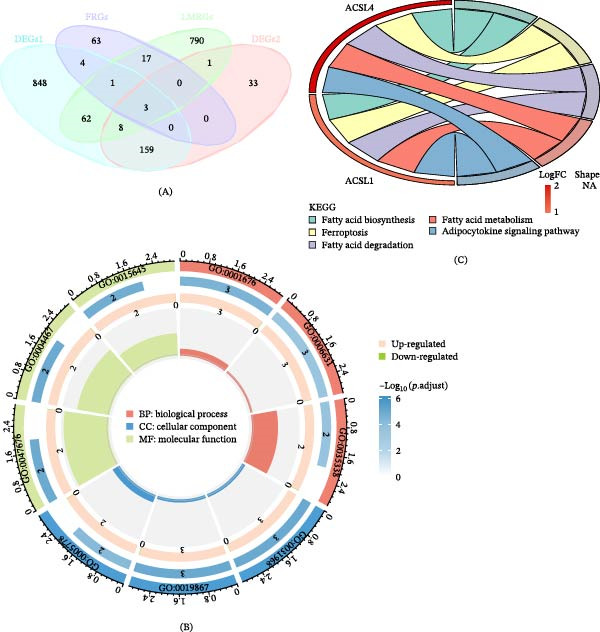
Identification analysis and functional enrichment of the three candidate genes. (A) Venn diagram showing the intersection of DEGs1, DEGs2, LMRGs (*n* = 882), and FRGs (*n* = 88), identifying three candidate genes. (B) GO enrichment analysis of the three candidate genes. (C) KEGG pathway enrichment analysis of the three candidate genes. Enrichment analyses in (B) and (C) were performed using the ClusterProfiler package, with a significance threshold of adjusted *p*‐value < 0.05.

The intersection of DEGs1, DEGs2, and LMRGs with FRGs revealed three intersected genes designated as the candidate genes for the subsequent analyses (Figure 2A). GO and KEGG analyses were performed on the three candidate genes to elucidate their functional enrichment. Enrichment analysis uncovered 299 enriched GO terms, including 259 BPs, 18 CCs, and 22 MFs, together with 29 KEGG pathways (Figure 2B,C, Tables S3,S4). Specifically, the most relevant enriched terms were long‐chain fatty acid metabolic process and fatty acid metabolic process (BP), organelle outer membrane and outer membrane (CC), arachidonate‐CoA ligase activity and long‐chain fatty acid‐CoA ligase activity (MF), and fatty acid biosynthesis and ferroptosis (KEGG).

### 3.3. Identification of Two Biomarkers and Their Distinguishing Sample Performance

**Figure 3:**
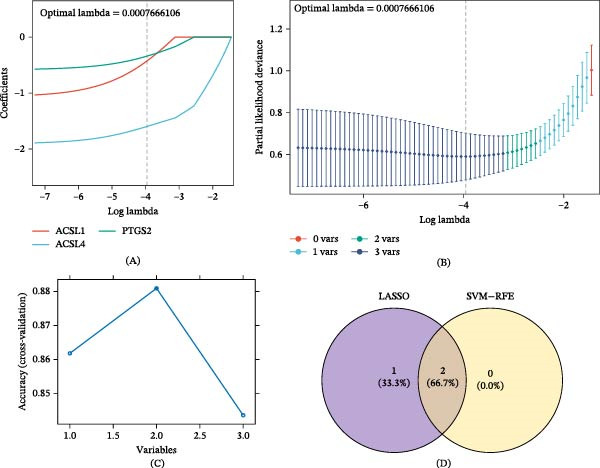
Identification of candidate biomarkers. Analyses were based on the expression of the three candidate genes in the GSE87466 dataset (*n* = 108 samples). (A) LASSO coefficient profiles. (B) LASSO cross‐validation error curve; the vertical dashed line indicates the optimal lambda (lambda.min). The model employed 10‐fold cross‐validation. (C) SVM‐RFE feature selection accuracy plot. (D) Venn diagram identifying the final biomarkers (*ACSL4* and *PTGS2*) from the intersection of the LASSO and SVM‐RFE results.

**Figure 4:**
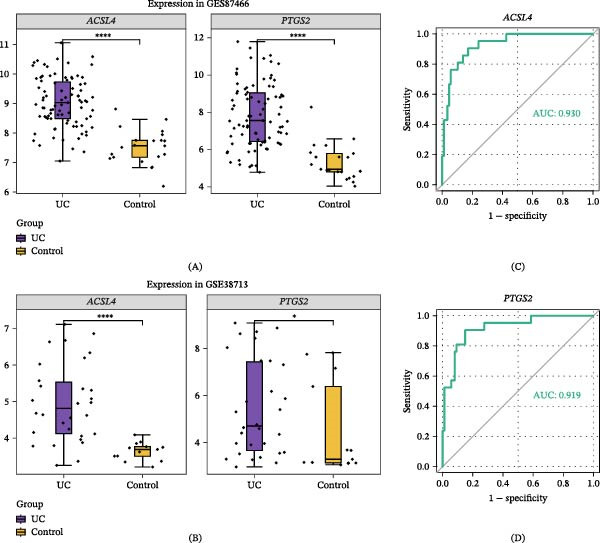
Identification of biomarkers and their sample differentiation performance. (A, B) Expression validation of *ACSL4* and *PTGS2* in the (A) GSE87466 (*n* = 108 samples, UC = 87, and control = 21) and (B) GSE38713 (*n* = 43 samples, UC = 30, and control = 13) datasets. Expression differences between UC and control groups were assessed using the Wilcoxon test (*p*  < 0.05). Data are presented as boxplots. (C, D) Receiver operating characteristic (ROC) curves demonstrating the diagnostic efficacy of (C) *ACSL4* (AUC = 0.930) and (D) *PTGS2* (AUC = 0.919) in the GSE87466 dataset.

Using the minimum error criterion, the LASSO logistic regression model identified λ_min = 0.0007666106 as the point of lowest model error and retained three genes: *ACSL4*, *PTGS2*, and *ACSL1* (Figure 3A,B). When the number of candidate genes was two, the SVM‐RFE model had the highest prediction accuracy, with *ACSL4* and *PTGS2* as the predicted genes (Figure 3C). The genes selected based on both LASSO and SVM‐RFE served as candidate biomarkers (Figure 3D). Wilcoxon test analysis uncovered that *ACSL4* and *PTGS2* (the two candidate biomarkers) displayed statistically significant expression discrepancies between UC and control samples in both datasets (GSE87466 and GSE38713; *p*  < 0.05), with a prominent upregulation of these two genes in the UC cohort (Figure 4A,B). These genes were used as biomarkers for subsequent analyses. ROC curve analysis demonstrated that *ACSL4* (AUC = 0.930) and *PTGS2* (AUC = 0.919) effectively discriminated between UC and control samples in the GSE87466 dataset (Figure 4C,D).

### 3.4. GSEA of Two Biomarkers and Immune Infiltration Analysis of UC

**Figure 5:**
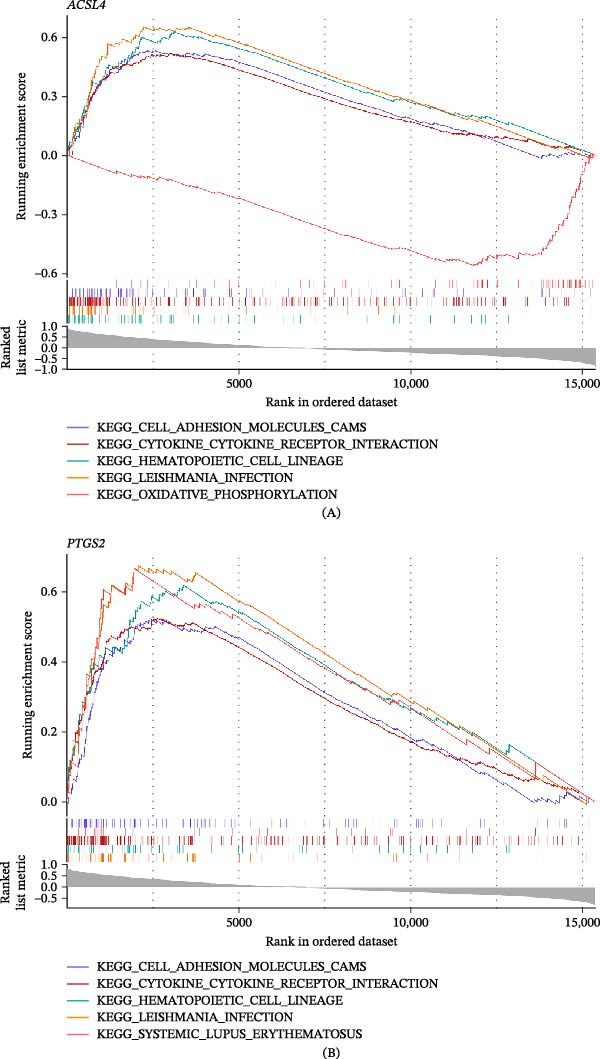
GSEA of biomarkers (A–B): GSEA enrichment analyses of *ACSL4* and *PTGS2*. (A and B) Top five KEGG pathways from leading‐edge subset analysis of enriched genes ranked by correlations with *ACSL4* and *PTGS2* expression, respectively, in GSE87466 (UC and control samples, *n* = 108). GSEA was performed using the c2.cp.kegg.v7.4.symbols.gmt gene set. Pathways with a normalized enrichment score (NES) absolute value > 1 and an adjusted *p*‐value (*p*.adjust) < 0.05 were considered significantly enriched.

According to the threshold of *p*.adjust < 0.05 and |NES| > 1, 79 KEGG pathways were significantly enriched in association with *ACSL4*, including hematopoietic cell lineage, cytokine–cytokine receptor interaction, and oxidative phosphorylation (Figure 5A and Table S5), and 78 KEGG pathways were significantly enriched in association with *PTGS2*, including hematopoietic cell lineage, cytokine–cytokine receptor interaction, and systemic lupus erythematosus (SLE) (Figure 5B and Table S6). These findings suggest that *ACSL4* and *PTGS2* jointly regulate hematopoietic cell lineage, Leishmania infection, cell adhesion molecules (CAMs), and cytokine–cytokine receptor interaction pathways involved in UC development.

In the GSE87466 dataset, 27 immune cell types differed significantly between the UC and control cohorts, including activated B cells, activated CD4 T cells, activated CD8 T cells, activated dendritic cells, CD56bright natural killer cells, and CD56dim natural killer cells (Figure 6A,B). The correlation results indicated that *ACSL4* was significantly negatively associated with CD56dim NK cells (*R* = −0.4624 and *p*  < 0.05)—a similar negative correlation existed between *PTGS2* and CD56dim NK cells (*R* = −0.4110 and *p*  < 0.05) (Figure 6C and Table S7). Significant positive associations were detected between the biomarkers and other differentially expressed immune cells, excluding CD56dim natural killer cells (|*R*| > 0.3 and *p*  < 0.05). Collectively, these results suggest that biomarkers are involved in UC pathogenesis through their coordinated variations with a subset of immune cells.

Figure 6Biomarker‐associated immune cells. (A) Heatmap of immune cell infiltration in the GSE87466 dataset (*n* = 108). (B) Boxplots of differential immune cell infiltration between the control group (*n* = 21) and UC group (*n* = 87), compared using the Wilcoxon test (*p*  < 0.05). (C) Bar plot of correlations between biomarkers and differential immune cells, assessed by Spearman’s correlation (|*R*| > 0.3, *p*  < 0.05) (*x*‐axis: correlation coefficient; positive values indicate positive correlation, negative values indicate negative correlation, and zero indicates no correlation; *y*‐axis: different immune cells).

**Figure:**
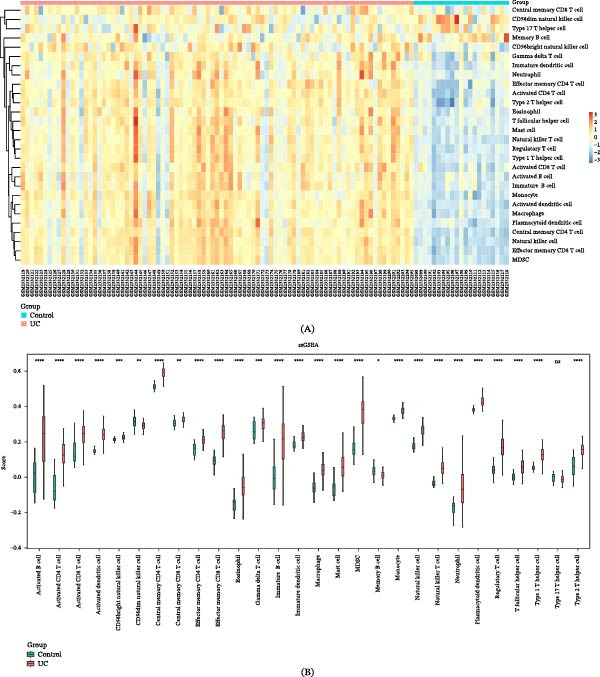

**Figure:**
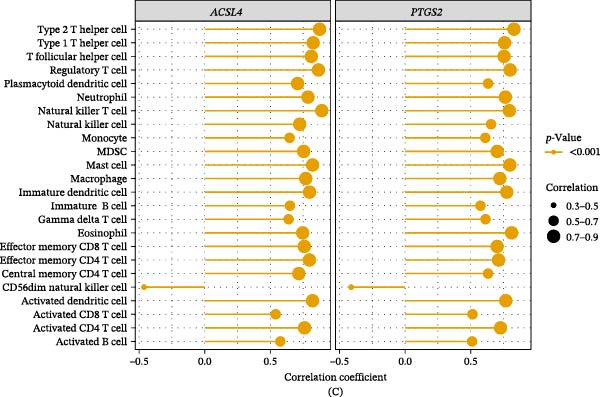

### 3.5. Regulatory Networks of Two Biomarkers

We predicted that 26 miRNAs are involved in regulating the two biomarkers: hsa‐miR‐106a‐5p, hsa‐miR‐130a‐3p, hsa‐miR‐17‐5p, and additional miRNAs modulated *ACSL4*; hsa‐miR‐101‐3p, hsa‐miR‐26a‐5p, hsa‐miR‐576‐5p, and others regulated *PTGS2*. Thereafter, seven lncRNAs were retrieved from the 26 miRNAs, with MALAT1 exhibiting binding affinity for hsa‐miR‐576‐5p and hsa‐miR‐503‐5p (Figure 7A and Table S8). Furthermore, 11 TFs were predicted to target *ACSL4* (e.g., PRRX2, GATA2, and CREB1) and six TFs to target *PTGS2* (e.g., CREB1, GATA2, and FOX1), with GATA2 and CREB1 identified as shared coregulators of *ACSL4* and *PTGS2* (Figure 7B).

Figure 7miRNA–lncRNA pairs and TFs associated with biomarkers. (A) Sankey diagram visualizing the ceRNA network, depicting the interactions among lncRNAs, miRNAs, and biomarker genes (mRNAs). The miRNA–mRNA interactions were predicted using the multiMiR package (confidence threshold > 0.5), and lncRNA–miRNA interactions were retrieved from the starBase database. (B) Network diagram visualizing the TFs predicted to regulate *ACSL4* and *PTGS2*. TFs were predicted using the JASPAR database via the NetworkAnalyst platform. Shared regulators (GATA2 and CREB1) are highlighted.

**Figure:**
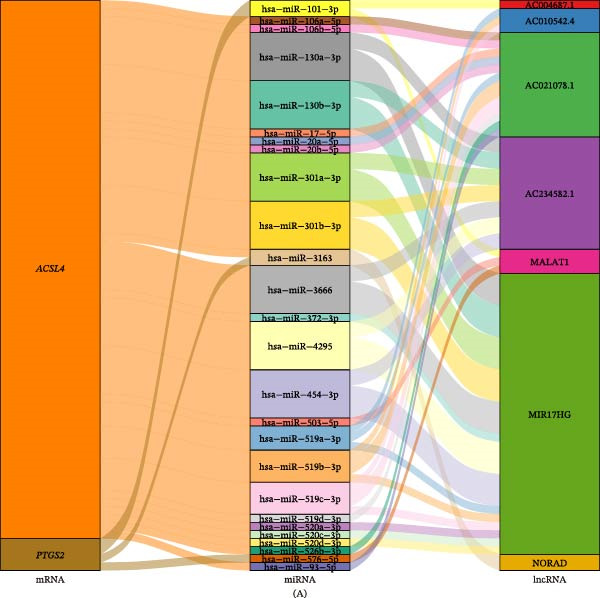

**Figure:**
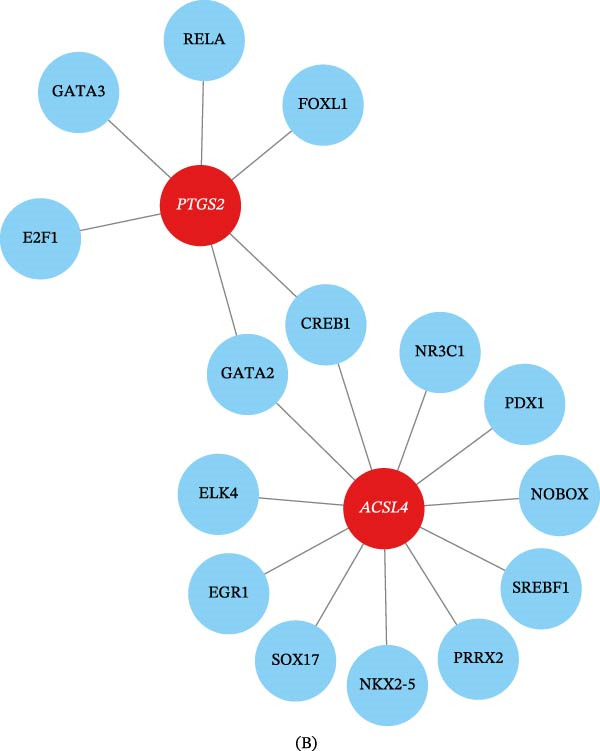

### 3.6. Identification of 41 Predicted Drugs

**Figure 8:**
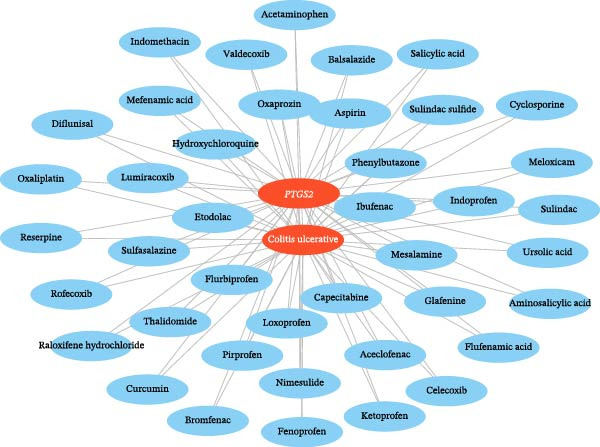
Network analysis of 41 drugs predicted to interact with UC and *PTGS2*. Drug–UC associations were sourced from CTD, and drug–*PTGS2* interactions from DGIdb. The network was constructed using Cytoscape, with first‐line UC therapies highlighted.

In this study, 41 drugs targeting UC and *PTGS2* were predicted using CTD (https://ctdbase.org/) and DGIdb (https://www.dgidb.org/), including sulfasalazine, celecoxib, and mesalamine (Figure 8 and Table S9).

### 3.7. Verification of Two Biomarkers in Clinical Samples

**Figure 9:**
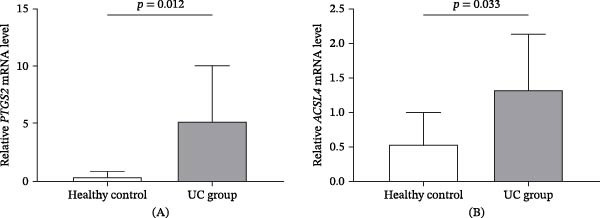
Validation of two biomarkers in clinical samples. (A) Analysis of *PTGS2* expression levels in UC (*n* = 10) and control samples (*n* = 5). (B) Relative ACSL4 expression (mean ± SD) was quantified in triplicate RT‐qPCR assays using the 2−ΔΔCt method with a housekeeping gene control. Group comparisons used an independent samples *t*‐test ( ^∗^ *p*  < 0.05).

RT‐qPCR profiling experiments revealed that both biomarkers displayed distinct expression signatures in patients with UC (*n* = 10) versus healthy controls (*n* = 5) (Figure 9). Tables S10 and S11 present the results of the sample gene Ct values and RQ values. Relative to the control group, *PTGS2* mRNA expression levels were notably augmented in patients with UC, and this disparity was statistically significant (*p* = 0.012) (Figure 9A). The UC cohort exhibited a statistically significant elevation in *ACSL4* expression relative to the controls (*p* = 0.033) (Figure 9B). Both genes showed statistically significant upregulation in the UC samples, consistent with the bioinformatics analysis results from the GSE87466 and GSE38713 datasets.

## 4. Discussion

As a chronic IBD targeting the colon, UC has been identified as a key predisposing factor for colon carcinoma onset [45]. Intestinal epithelial cell damage constitutes a critical component of UC tissue injury. Ferroptosis is associated with intestinal epithelial cell death in UC [46]. Interestingly, the significant accumulation of membrane lipid peroxides characterizes ferroptosis. Therefore, enhancing or inhibiting iron oxidation by regulating lipid metabolism may be a new approach for treating UC. In this study, we conducted bioinformatics analysis on patients with UC based on the GEO public database and obtained two biomarkers (*ACSL4* and *PTGS2*), which were potential genes associated with lipid metabolism‐mediated ferroptosis in UC.

*ACSL4* belongs to the five‐member ACSL family. It is a key enzyme in lipid metabolism and catalyzes the conversion of long‐chain fatty acids, especially polyunsaturated fatty acids (PUFAs), into acyl‐CoA esters. This process is crucial for phospholipid biosynthesis and ferroptosis execution [47]. *ACSL4* comprises an N‐terminal domain, two luciferase‐like regions, a ligand that connects the two luciferase‐like regions, and a C‐terminal domain, with the latter two regions being crucial for its enzymatic activity [48]. Notably, *ACSL4* shows strong substrate preference for 20‐carbon PUFAs such as arachidonic acid (AA) and adrenic acid, which are key precursors of lipid peroxidation—the hallmark event of ferroptosis [49]. In UC, the dextran sulfate sodium–induced colitis model showed elevated *ACSL4* expression. Inhibiting its activity through ferroptosis inhibitors such as ferrostatin‐1 or vitamin D treatment markedly alleviates disease severity, indicating that *ACSL4*‐mediated ferroptosis plays a pathogenic role in UC [50–52]. *ACSL4* upregulation in patients with UC drives pathology by dysregulating enriched pathways. In hematopoietic cell lineage, *ACSL4* may alter lipid metabolism, promoting proinflammatory macrophage polarization and suppressing regulatory T cell development, thereby exacerbating chronic inflammation [53]. For cytokine–cytokine receptor interaction, *ACSL4* may enhance lipid‐derived mediator synthesis (e.g., AA metabolites), amplifying cytokine signals (e.g., TNF‐α and IL‐6) and leading to sustained immune activation and epithelial barrier damage in the colon [54]. Given *ACSL4*’s dual function in lipid metabolism and ferroptosis, this enzyme has become a highly promising therapeutic target for UC, and future interventions may restore intestinal homeostasis by modulating its activity.

*PTGS2*, a gene encoding cyclooxygenase‐2 (COX‐2), is considered a typical potential biomarker for cells undergoing ferroptosis, with its expression significantly upregulated during iron‐dependent cell death [55]. From a mechanistic perspective, *PTGS2* catalyzes the conversion of AA into prostaglandin E2 (PGE_2_), a key inflammatory mediator that exacerbates oxidative stress and lipid peroxidation, both of which are central drivers of ferroptosis. Triggering the COX‐2/PGE_2_ signaling pathway can counteract the elevated expression of miR‐137, a microRNA that protects cells from lipid peroxidation and iron overload, which ultimately induces ferroptosis [56]. *PTGS2* also plays a key role in chronic inflammatory diseases. In rheumatoid arthritis, *PTGS2* expression in synovial cells increases, culminating in the production of large amounts of PGE_2_, which causes joint inflammation, pain, and swelling [57, 58]. Similar phenomena have also been observed in psoriasis—*PTGS2* upregulation promotes abnormal proliferation of keratinocytes and immune dysregulation [59]—and in chronic spontaneous urticaria, which is closely related to mast cell activation and inflammatory response [60]. *PTGS2*’s dual role in ferroptosis and inflammation makes it an important therapeutic target, and COX‐2 inhibitors (such as nonsteroidal anti‐inflammatory drugs) have been widely used to manage inflammatory diseases. Future research should explore whether targeting the *PTGS2*‐mediated ferroptosis pathway can provide novel treatment strategies for inflammatory diseases such as UC.

GSEA shows that *ACSL4* and *PTGS2* are closely linked to key pathways in UC progression. With notable enrichment in the hematopoietic cell lineage and the cytokine–cytokine receptor interaction pathway, *ACSL4* is proposed to participate in disease progression by modulating immune cell differentiation processes and inflammatory signaling pathways. The interaction between cytokines and their specific receptors triggers downstream signaling pathways, culminating in the release of proinflammatory mediators and the recruitment of immune cells to intestinal regions exhibiting inflammation [61]. Given that hematopoietic system disorders can promote chronic intestinal inflammation, *ACSL4* may affect UC pathogenesis by altering immune cell populations and cytokine‐mediated responses [62]. This gene is also associated with the oxidative phosphorylation pathway, further confirming its involvement in metabolic reprogramming and ferroptosis—mitochondrial dysfunction and pathological elevation of reactive oxygen species levels are key drivers of UC epithelial cell death [63]. Similarly, *PTGS2* is highly concentrated in hematopoietic cell lineage and cytokine–cytokine receptor interaction pathways, reinforcing its role in immune dysregulation. This gene is also closely associated with SLE and CAM pathways, suggesting potential interactions between UC and autoimmune mechanisms. Prostaglandins derived from *PTGS2*, such as PGE_2_, can regulate leukocyte adhesion and endothelial cell activation; upregulation of their expression may exacerbate mucosal barrier disruption and immune cell infiltration in patients with UC [57, 64]. Additionally, *PTGS2* enrichment in Leishmania infection pathways reveals common inflammatory cascades between infectious and autoimmune colitis triggers, highlighting the gene’s broad role in immune‐mediated tissue damage [60, 64]. These findings collectively indicate that *ACSL4* and *PTGS2* are associated with UC development through computational correlations with immune activation, metabolic stress, and ferroptosis, thus providing hypotheses for therapeutic intervention. The analyses presented in Figures 4–8 (including biomarker identification, GSEA, immune infiltration, regulatory networks, and drug prediction) serve as a cohesive computational framework supporting the main claim that *ACSL4* and *PTGS2* are key biomarkers linked to lipid metabolism–ferroptosis in UC. However, these are associative insights derived from bioinformatics, and future experimental studies are needed to validate causal mechanisms.

Our study identified significant changes in 27 immune cells between patients with UC and control groups using the GSE87466 dataset, including activated B cells, CD4^+^/CD8^+^ T cells, dendritic cells, and CD56bright/dim NK cell subpopulations, revealing deep dysregulation of innate and adaptive immune systems in UC pathogenesis. A hallmark of IBD is the abnormal activation of gut innate and adaptive immune responses. Inflammatory mediators secreted by activated innate immune cells act as key drivers of adaptive immune activation, and they further govern disease progression by shaping the differentiation of CD4 T cells into specialized subsets (i.e., Th1, Th2, Th17, Tfh, and Treg) [65]. Particularly, CD8^+^ effector T cells derived from patients with UC drive tissue injury by generating TNF‐α [66]. Conversely, existing research has poorly characterized B cell contributions to UC pathogenesis; Uzzan et al. [67] reported a markedly dysregulated B cell response and suggested a potential pathogenic role. NK cell compartments exhibit special complexity: The CD56dim subset exerts cytotoxic effects, while the CD56bright subset has immunoregulatory characteristics [68]. *ACSL4*’s negative correlation with CD56dim NK cells in UC indicates a potential immunosuppressive mechanism. High *ACSL4* expression may suppress NK cell cytotoxicity (via the PUFA metabolism network) [69], impairing epithelial clearance and sustaining inflammation, thereby exacerbating mucosal injury and tissue repair dysfunction. Recent single‐cell epigenomic studies have further revealed heterogeneous histone modification patterns in IBD‐derived CD56bright NK cells, suggesting that epigenetic mechanisms regulate their functional plasticity [70]. These findings collectively characterize UC as a multidimensional immune dysregulation disease: Abnormal interactions between lymphocyte subsets form self‐sustaining inflammatory microenvironments, ultimately leading to chronic intestinal damage. The identification of these cell‐specific changes advances understanding of UC immunopathology and offers potential cellular targets for precision immunotherapy.

The identified miRNAs contribute to UC pathophysiology by fine‐tuning biomarker expression. Dysregulation of these miRNAs might disrupt lipid metabolism and immune balance, exacerbating UC progression. Their interaction with lncRNAs (e.g., MALAT1) further adds complexity to the regulatory network.

Our drug predictive analysis found that first‐line UC treatment drugs such as sulfasalazine and mesalamine can serve as potential *PTGS2*‐targeting agents, providing validation for the mechanistic rationale of bioinformatics methods. Notably, mesalamine exerts therapeutic effects through multiple anti‐inflammatory mechanisms. While it directly reduces mucosal prostaglandin (PGE_2_) and leukotriene (LTB_4_) production by inhibiting COX (including *PTGS2*/COX‐2) and lipoxygenase activity, it inhibits the release of cytokines (TNF‐α, IL‐1β, and IL‐6) mediated by NF‐κB [71, 72]. This dual inhibition of the eicosanoid and cytokine pathways synergistically reduces immune cell infiltration by impeding T cell activation and neutrophil recruitment to the intestinal mucosa. These clinically effective drugs align highly with our predicted *PTGS2*‐targeting agents, confirming PTGS2’s biological significance in UC pathogenesis and indicating that our computational drug prediction process can identify candidate drugs with a mechanistic basis. These findings provide theoretical support for repurposing existing COX inhibitors or developing next‐generation *PTGS2*‐selective inhibitors, which may become a key component of precision medicine strategies for UC.

## 5. Conclusion

This study systematically identified *ACSL4* and *PTGS2* as key markers of the lipid metabolism–ferroptosis axis in UC through integrated multiomics analyses, revealing their molecular mechanisms for promoting disease progression through cytokine network dysregulation and immune microenvironment remodeling. This provides an important theoretical basis for the precise diagnosis and treatment target development of UC. However, this study has certain limitations: The relatively limited sample size of retrospective cohorts may affect statistical validity, and transcriptome analysis based on public databases may not fully reflect the true changes in protein levels. Hence, future research should focus on the following directions: first, conduct multicenter prospective cohort studies to synchronously detect the dynamic correlation between *ACSL4*/*PTGS2* protein expression and clinical activity through ELISA or mass spectrometry flow cytometry technology; second, using organoid models or conditional gene knockout mice, the functional mechanisms of these two targets and the feasibility of their intervention strategies can be validated in a simulated human intestinal microenvironment, and verify whether *ACSL4* and *PTGS2* can further subdivide subtypes, thereby connecting molecular heterogeneity with clinical phenotypes, which will lay a more solid experimental foundation for translational medicine research. These follow‐up works will help improve the theoretical system of this study and promote the clinical translation process of individualized treatment plans for UC.

## Funding

This study was funded by the Development Plan of Traditional Chinese Medicine Science and Technology in Jiangsu Province (Grant QN202217).

## Conflicts of Interest

The authors declare no conflicts of interest.

## Supporting Information

Additional supporting information can be found online in the Supporting Information section.

## Supporting information


**Supporting Information** Table S1: 882 LMRG functional groups. Table S2: 88 FRGs. Table S3–S4: GO and KEGG enrichment pathways of candidate genes. Table S5–S6: GSEA enrichment pathways of ACSL4 and PTGS2. Table S7: Correlation *p*‐values and coefficients between biomarkers and differential immune cells. Table S8: miRNA–lncRNA pairs associated with biomarkers. Table S9: Drugs related to UC and biomarkers. Table S10: Ct value and RQ value of ACSL4. Table S11: Ct value and RQ value of PTGS2.

## Data Availability

The data that support the findings of this study are available in the supporting material of this article. In the experimental design and data collection process of this study, no cell lines were involved. The relevant research work was completed based on population epidemiological data (e.g., analysis of the NHANES database) and clinical sample testing. Therefore, there is no need to provide information related to cell lines.
